# Editorial: AI and Financial Technology

**DOI:** 10.3389/frai.2019.00025

**Published:** 2019-11-15

**Authors:** Paolo Giudici, Ronald Hochreiter, Jörg Osterrieder, Jochen Papenbrock, Peter Schwendner

**Affiliations:** ^1^Department of Economics and Management, University of Pavia, Pavia, Italy; ^2^Department of Business and Management, Webster Vienna Private University, Vienna, Austria; ^3^School of Engineering, Zurich University of Applied Sciences, Winterthur, Switzerland; ^4^Firamis GmbH, Frankfurt, Germany; ^5^Center for Asset Management, School of Management and Law, Zurich University of Applied Sciences, Winterthur, Switzerland

**Keywords:** FinTech, SupTech, RegTech, AI, machine learning, P2P lending, Blockchain

The Financial Stability Board defines FINancial TECHnology as “technologically enabled financial innovation that could result in new business models, applications, processes, or products with an associated material effect on financial markets and institutions and the provision of financial services.” While innovation in Finance is not a new concept, the focus on technological innovations and its pace have increased significantly. Fintech solutions that make use of Big Data analytics, Artificial Intelligence, and Blockchain technologies are currently introduced at an unprecedented rate. These new technologies are changing the nature of the financial industry, creating opportunities for Fintechs startups to offer more inclusive access to financial services. The advantages notwithstanding, Fintech solutions leave the door open for many challenges such as underestimation of creditworthiness, market volatility, cyber attacks, fraud and money laundering which represent central points of interest for regulators and supervisory bodies.

In this context, a key issue becomes identifying the desired level of trade-off between innovation incentives on one hand, and mitigation of risks on the other. The European regulatory framework should enable Fintech companies operating in their jurisdiction to benefit from innovations in Technology and Finance while at the same time ensuring both a high level of protection for consumers and investors and resilience of the financial system. This point has been framed by the current European Commissioner for the Euro and Social Dialogue and Vice-President of the European Commission, Valdis Dombrovskis: “Across the board, we are working to strike the right balance between risks and opportunities; so that Europe can benefit fully from new technologies in the financial services sector.”

There is a strong need to improve the competitiveness of the European Fintech sector, introducing a framework for a common regulatory approach across all countries that can supervise Fintech companies without stifling their economic potential. Such a framework should support both Fintechs as well as supervisors: on one hand, Fintech firms that want to grow and scale-up across Europe require a neutral technology and proportional regulatory compliance as well as advice on how to identify opportunities for innovation procurement, e.g., in advanced regulatory technology (RegTech) solutions; on the other hand, the supervisory bodies' ability to monitor innovative financial products proposed by Fintechs is limited and advanced supervisory technology (SupTech) solutions are required.

The Horizon 2020 project FIN-TECH (Financial Supervision and Technological Compliance)—funded by the European Commission for the period 2019–2020—conducts research on Fintech risk management models to be shared with European regulators, Fintechs as well as banks. These models are evaluated on a global level which helps to close the gap between technical and regulatory expertise, in particular providing risk management procedures common to both sides and uniform across countries. It will eventually lead to the development of a regulatory framework that encourages innovations in Big Data analytics, Artificial Intelligence, and Blockchain technologies which, at the same time, satisfies supervisory concerns to apply regulations in an effective and efficient way, that well protects consumers and investors. In particular, the FIN-TECH project aims to create a European training program aimed at shared risk management solutions that automatize compliance of Fintech companies (RegTech) and, at the same time, increases the efficiency of supervisory activities (SupTech). In other words, the project aims at connecting FINancial supervision with TECHnological compliance.

This special issue contains the first contributions from this European project. Some of them are research papers that evolved into use cases of the project and are shared as well as used by regulators, banks, and Fintechs. Other papers are based on extensive talks given by external speakers that participated at specific events organized by the project. This collection of papers is discussing public policy viewpoints as well as AI applications to measure market risks and credit risks especially in the areas of Robo Advisory and Peer to Peer (P2P) lending.

The paper by Bredt as well as the paper by O'Halloran and Nowaczyk present and discuss public policy strategies aimed at addressing financial innovations brought by disruptive technologies, capturing their opportunities while mitigating the related risks.

Furthermore, the paper by Schwendner et al., the paper by Hakala and the paper by Pagnottoni show how Machine Learning methods and Artificial Intelligence solutions can be employed to develop new asset management practices addressing risks. While Schwendner et al. focus on European Bonds, Hakala considers modeling volatilities and Pagnottoni works on Blockchain based bitcoin transactions.

The paper by Giudici et al. the paper by Ahelegbey et al., and the paper by Agosto et al. all consider how the measurement of network effects arising from Peer to Peer (P2P) lending platforms can improve the measurement of credit risk of borrowers. They apply different models but use the same database with the common goal to provide applicable use cases for the FIN-TECH European project to monitor and control credit risk arising from the application of Big Data analytics.

Finally, the paper by Agosto and Raffinetti focuses on building appropriate model comparison tools for credit risk modeling.

## Author Contributions

All authors listed have made a substantial, direct and intellectual contribution to the work, and approved it for publication.

### Conflict of Interest

JP was employed by the company Firamis GmbH. The remaining authors declare that the research was conducted in the absence of any commercial or financial relationships that could be construed as a potential conflict of interest.

